# Evaluation of dried papaya pomace meal in laying hen diets

**DOI:** 10.1002/vms3.516

**Published:** 2021-05-06

**Authors:** Bogale Tamiru, Ashraf Alkhtib, Metekia Tamiru, Solomon Demeke, Emily Burton, Taye Tolemariam, Lemessa Debela, Geert P. J. Janssens

**Affiliations:** ^1^ College of Agriculture and Veterinary Medicine Department of Animal Science Jimma University Jimma Ethiopia; ^2^ School of Animal, Rural and Environmental Sciences Nottingham Trent University Brackenhurst Campus Nottingham UK; ^3^ Department of Biomedical Science Institute of Health Science Jimma University Jimma Ethiopia; ^4^ Faculty of Veterinary Medicine Department of Nutrition, Genetics and Ethology Ghent University Merelbeke Belgium

**Keywords:** *carica papaya*, haematology, laying performance, peel, seed

## Abstract

In the search for alternative feed resources for laying hens, papaya pomace is available as industrial by‐product but information on its nutritive value is lacking. Dried papaya pomace was included in a common laying hen diet at 0%, 2.5%, 5% and 7.5% to evaluate its effect on egg production performance, egg quality and general health parameters in Bovan brown layers. For every inclusion level, three cages with ten 20‐week‐old layers were used, making a total of 120 hens. The effect of dried papaya pomace inclusion on egg production, egg quality and general health parameters was evaluated. Dried papaya pomace inclusion improved egg production and laying by 6.15% and 17% respectively, while it significantly decreased feed conversion ratio by 7.5%. Eggshell weight, thickness and strength of PP5 were higher than the control by 0.3 g, 0.8 mm and 0.43 kg/cm^2^, respectively. There was a significant improvement in albumin weight (by 1.5 g/L), albumin height (2.1 mm), yolk weight (0.4 g/L), yolk height (0.4 mm), yolk colour (4.4 points) and Haugh unit (8 points) due to PP5 treatment. Inclusion of papaya pomace at a level of 7.5% of layers diet had negative effect on Egg production, feed conversion ratio and interior and exterior egg quality traits. Inclusion of papaya pomace affected significantly serum total cholesterol, serum triglyceride, serum low density lipoprotein, serum high density lipoprotein, aspartate aminotransferase, alanine aminotransferase and white blood cell count. However, all blood traits of the experimental animals were within the normal ranges reported for layers. Inclusion of papaya pomace in 5% of layers commercial diets improves egg production and quality without negative impact on health while decreasing feeding cost.

## Abstract 2

Payaya pomace inclusion at a level of 5% improved layering hens’ performance. Payaya pomace inclusion improved serum biochemical profile of layer chicken. Payaya pomace inclusion didn’t affect hematological profile of laying chicken.

## 1 INTRODUCTION

Conventional feed ingredients that are in short supply are generally costly in developing countries and contribute largely to the increase in poultry production costs. Poultry nutritionists in developing countries have therefore prioritized to introduce local non‐conventional feed stuffs, such as fruit production and processing by‐products (Kasapidou et al., 2015), into poultry diets. Mango (Emshaw et al., 2012), guava (Lira et al., 2009) and apple (Ayhan et al., 2009) already proved to be cheap and suitable sources of nutrients for poultry. Moreover, extensive studies have been carried out to unveil the potential of varieties of seed meals for poultry including blue lupin (Al‐Sagan et al., 2020), cumin (Saleh et al., 2020), flaxseeds and/or fenugreek seeds (Saleh et al., 2019), pumpkin seed (Martínez et al., 2010), sunflower seed (Rama Rao et al., 2006) and cotton seed (Swiatkiewicz et al., 2016). Variety of herbal plants have also been explored in poultry nutrition to improve productivity, immune response and health status (Aroche et al., 2018); (Haselmeyer et al., 2014); (Saleh et al., 2018); (Navid et al., 2014); (Gerzilov et al., 2015); (Saki et al., 2014).

Papaya (*Carica papaya*) is a plant native to tropical America and southern Mexico and currently planted in Brazil, India, Mexico and Africa (Gha et al., 2019). Papaya is one of the most popular fruits in Ethiopia grown for its popular taste and high nutritive value. Ethiopia produces annually 54,355 tons of papaya fruits (Gabrekiristos, 2020); (Setargie et al., 2015). Direct consumption and juice extraction of papaya fruits result in seeds, peels and small amount of pulp and this combination is considered as pomace. A large volume of papaya pomace is produced in Ethiopia annually (Saleh et al., 2019). On dry matter basis, literature reports that papaya pomace contains a fairly high crude protein concentration (179–184 g/kg) and still quite some energy (17‐MJ gross energy/kg) (Martínez et al., 2010); (Rama Rao et al., 2006). In addition, papaya peel contains functional compounds including proteolytic enzymes (papain and chymopapain) and anthelmintic, antibacterial and anticoccidial compounds (Diarra, 2018); (Pavithra et al., 2018).

Some studies have already evaluated papaya by‐products in broilers for instance papaya seed (Sola‐ojo & Bolu 2009), papaya peel (Saleh et al., 2018). To our knowledge, papaya pomace has not been evaluated as feed ingredient for laying hens. The goal of our study was therefore to evaluate a range of dietary inclusion levels of dried papaya pomace on egg production, egg quality and general health in Bovan brown layers.

## 2 MATERIAL AND METHODS

### 2.1 Feeding and set‐up

One hundred twenty 20‐week‐old Bovan brown layers were obtained from Alema Poultry PLC, Debre Zeit, Ethiopia, weighed and randomly allocated into 12 cages, with 10 birds per cage (100 cm × 90 cm × 90 cm) in a house having similar environmental condition. The average relative humidity and temperature of the outside environment was 78% and 20°C respectively which depended the house microclimate. The cages were randomly allotted to four inclusion levels of dried papaya pomace: 0%, 2.5%, 5% and 7.5%. Standard layers commercial mashed concentrate formulated to meet the nutrient requirement of Bovan brown at initial age of 20 weeks were obtained from Alema feed processing company.

Papaya pomace was collected from local juice shops in Jimma town, mixed thoroughly, naturally dried in a shed, ground using a mortar mill with a sieve size of 4 mm and properly stored. The processed meal was packed in bags of 100 kg and stored in a dry place until required. As indicated in Table 1, the diets contained white maize, toasted soybean, vitamin/mineral premixes, meat and bone meal, wheat bran, noug seed cake (*Guizotia abyssinica*), dried papaya pomace meal, limestone flour and salt. Four iso‐nitrogenous and isocaloric treatments were made using the above‐mentioned ingredients which were ground with similar sieve size separately before mixing. Except for the control treatment, toasted soybean meal and noug seed cake (*G. abyssinica*) were substituted by dried papaya pomace meal, and uniformly mixed. Then, the mixed ingredients were further uniformly mixed with other ingredients. The dietary treatments were the control diet and diets containing dried papaya pomace meal at the levels of 2.5%, 5%, and 7.5% partially substituting the protein of the toasted soybean and noug cake (*G. abyssinica*) in the control diet. Proximate analysis was performed to identify the chemical composition of dietary treatments including dried papaya pomace meal.

**TABLE 1:** Chemical composition (% dry matter) and metabolizable energy content of the experimental feeds

	DM	CP	EE	ASH	CF	NFE	ME
Papaya pomace	90	19.2	4.5	18	28.6	29.7	12.2

Abbreviations: CF, crude fiber; CP, crude protein (% DM); DM, dry matter; EE, ether extract (%DM); ME, metabolizable energy (MJ/kg DM) calculated according to Wiseman (1987); NFE, nitrogen free extract (% DM).

Before the start, birds were vaccinated against Newcastle disease, fowl typhoid, fowl pox, infectious bursal disease and Marek's disease as per recommended for Bovan brown layers. Birds were offered the experimental diets *adlibitum* and feed intake was calculated by subtracting the leftover feed from the offered feed. All experimental birds had free access to clean drinking water. The trial lasted for 12 weeks in addition to one week of adaptation.

### 2.2 Laying performance and egg quality

Individual weights of the birds were recorded at start and end (12 weeks) of the trial. Egg production, egg weight, and feed intake, egg mass, feed conversion ratio, egg production and laying rate were recorded for each replicate (cage). Three eggs were randomly collected from each cage every week to determine egg interior and exterior quality. Shell weight, shell strength (measured by an egg force reader [EFR‐01; Orka Food Technology Ltd]) and shell thickness (determined by eggshell thickness gauge (Robotmation Co., Ltd.) at the large end, equatorial region, and small end) were determined. A Roche colour fan (Hoffman‐La Roche Ltd., Basel, Switzerland) was used to determine egg yolk colour. Albumin and yolk weights were recorded while yolk height wasmeasured using an electronic digital calliper (SH14100025; Shenhan, Shanghai, China). Haugh unit was calculated according to Card and MCN (Card et al., 1972).

### 2.3 Feed and blood analyses

Representative samples of the feeds were ground through passing a 1‐mm screen and then analysed for dry matter, ash, crude protein, ether extract and crude fibre according to AOAC (AOAC, 2006). Total nitrogen was determined using micro‐Kjeldahl method and crude protein was computed as nitrogen ×6.25. Nitrogen‐free extract was calculated by deducting ether extract, crude fibre and crude protein from organic matter and metabolizable energy was computed according to Wiseman (Wiseman, 1987). All the feed analyses were carried out at Jimma University, Animal Nutrition and Post‐harvest and Food Science Technology Laboratory.

At the end of the experiment, two birds were randomly selected from each cage and blood sampled from the brachial vein in a tube without anticoagulant for separation of serum; then the samples were immediately centrifuged at 2,268*g* for 15 min and the obtained serum was stored at −20°C until further analysis. Serum was analysed spectrophotometrically for total protein, albumin, uric acid, total cholesterol, triglycerides, low‐density lipoprotein, high‐density lipoprotein, aspartate aminotransferase, alanine aminotransferase and creatinine using commercial kits (Wiener LaboratoriosS. A.I.C., Argentina).

An additional two birds per cage were bled from punctured brachial vein to collect 5 ml of blood in EDTA tubes for haematological assays (Bermudez and Stewart‐Brown, 2003).

Red blood cells, white blood cell, packed cell volume, haemoglobin and absolute counts of neutrophils and lymphocytes were recorded according to Jain (Jain, 1986, 2005). All blood samples were analysed at Jimma University Medical Physiology Laboratory and Universal Clinic Chemical Pathology Diagnostic Laboratory, Jimma, Ethiopia.

### 2.4 Statistical analysis

One‐way analysis of variance was used to determine effect of papaya pomace supplementation on egg production, quality, serum biochemical and haematological profile. Means of treatments were compared using least significance difference test at *p* < .05. All statistical analyses were done using Statistical Analysis System (SAS, 2012).

## 3 RESULTS

The composition of the dried papaya pomace is presented in Table 1, and Table 2 illustrates the chemical composition of experimental treatments.

**TABLE 2:** Ingredient composition and nutrient analysis of the experimental diets

Dried papaya pomace inclusion	0%	2.5%	5%	7.5%	
Maize grain	48	48	48	48	
Toasted soybean meal	20	19	17.8	16.2	
Noug^a^ seed cake	14	13.5	13.5	12.5	
Vitamin and mineral premix	1	1	1	1	
Meat and bone meal	2	2	2	2	
Salt	0.5	0.5	0.5	0.5	
Limestone flour	4.5	4.5	4.5	4.5	
Wheat bran	10	10	10	10	
Dried papaya pomace	0	2.5	5	7.5	
Nutrient composition					
Dry matter (%)	90.9	90.9	90.9	90.8	
Ash (%)	8.56	8.80	9.03	9.27	
Crude protein (%)	18.0	17.0.8	17.4	17.1	
Crude fibre(%)	6.64	7.19	7.74	8.29	
Ether extract (%)	6.62	6.57	6.51	6.46	
Nitrogen free extract (%)	62.8	62.0	61.1	60.3	
Metabolizable energy (kcal/kg)	14.1	14.1	14.0	14.0	

^a^ *Guizotia abyssinica*; vitamin premix per kg of diet: vitamin A: 2.7 mg; vitamin D3 : 0.05 mg; vitamin E: 18 mg; vitamin K3: 2 mg; thiamine: 1.8 mg; riboflavin: 6.6 mg; pantothenic acid: 10 mg; pyridoxine: 3 mg; cyanocobalamin: 0.015 mg; niacin: 30 mg; biotin: 0.1 mg; folic acid: 1 mg; choline chloride: 250 mg; antioxidant: 100 mg; Fe: 50 mg; Mn: 100 mg; Zn: 100 mg; Cu: 10 mg; I: 1 mg and Se: 0.2 mg.

Although papaya pomace inclusion had no effect on feed intake and egg output, feed conversion ratio decreased from 0% to the 2.5% and 5% inclusions and increased again with 7.5% inclusion (Table 3). Egg laying rate showed a negative response to papaya pomace inclusion, with the highest laying rates in the 2.5% and 5% inclusion.

**TABLE 3:** Body weight, egg production and egg quality of Bovan Brown layers fed diets with varying inclusion levels of dried papaya pomace meal

Parameters	Dried papaya pomace inclusion				*SEM*
	0%	2.5%	5%	7.5%	
Initial live weight (kg)	1.81	1.81	1.81	1.81	0.07
Final live weight (kg)	1.82	1.83	1.86	1.81	0.07
Feed intake (g/day)	97	98	101	97	4
Egg weight(g/day)	67	69	71	65	2
Feed conversion ratio (g feed/g egg)	2.23^a^	1.99^b^	1.99^b^	2.26^a^	0.07
Laying rate (%)	78.5^b^	87.5^a^	89.2^a^	79.4^b^	2.4
Shell weight (g)	8.8	8.9	9.1	8.9	0.4
Shell thickness (mm)	63	64	64	63	3
Shell strength (kg/cm2)	5.1	5.2	5.5	5.1	0.2
Albumin weight (g/L)	40.6	41	42.1	44.3	1.9
Albumin height (mm)	8.1^b^	8.6^b^	10.2^ab^	11.2^a^	0.5
Yolk weight (g/L)	16.6	16.5	17.0	17.7	0.8
Yolk height (mm)	17.9	17.8	18.3	18.2	0.8
Yolk colour	1.0^b^	3.4^ab^	5.4^a^	8.6^a^	0.4
Haugh unit (%)	86^b^	89^ab^	94^ab^	101^a^	4

Note

^ab^ Means in a row with different superscript are significantly different (*p* < .05).

Abbreviation: *SEM*, standard error of the mean.

Shell characteristics (weight, thickness and strength) remained unaffected.

Egg white and egg yolk traits all increased with increasing papaya pomace inclusion, but only significant for albumin height, yolk colour and Haugh units.

Papaya pomace inclusion gradually reduced cholesterol, low density lipoprotein and uric acid and increased high density lipoprotein (Table 4). The dietary treatment did not significantly alter triglyceride, albumin, total protein, aspartate amino transferase, alanine amino transferase and creatinine concentrations in blood serum.

**TABLE 4:** Serum clinical biochemistry of Bovan Brown layers fed diets with varying inclusion levels of dried papaya pomace meal

	Dried papaya pomace inclusion				*SEM*
	0%	2.5%	5%	7.5%	
Total cholesterol (mg/dl)	123^a^	113^ab^	109^ab^	103^b^	5
Triglycerides (mg/dl)	273	257	265	239	12
Low density lipoprotein (mg/dl)	119^a^	110^ab^	91^b^	92^b^	5
High density lipoprotein (mg/dl)	44^b^	48^ab^	52^ab^	54^a^	3
Albumin (mmol/L)	26.2	26.2	26.1	26.1	1.3
Protein (mmol/L)	40	43	43	43	2
Uric acid (mmol/L)	5.8^a^	5.4^a^	5.2^a^	4.2^b^	0.2
Aspartate amino transferase(IU/L)	143	143	138	137	7
Alanine amino transferase (IU/L)	287	284	276	252	12
Creatinine(mmol/L)	71	71	71	71	3

Note

^ab^ Means in a row different superscript are significantly different (*p* < .05).

Abbreviation: *SEM*, standard error of the mean.

Papaya pomace meal had no significant influence on packed cell volume, haemoglobin, monocyte count, neutrophil counts, red blood cell counts and white blood cell counts (Table  5).

**TABLE 5:** Haematology of Bovan Brown layers fed diets with varying inclusion levels of dried papaya pomace meal

	Dried papaya pomace inclusion				*SEM*
	0%	2.5%	5%	7.5%	
Packed cell volume (%)	24.9	24.9	24.9	24.9	1.2
Haemoglobin (g/dl)	8.2	8.2	8.1	8.2	0.4
White blood cell (×10^9^/L)	2.38	2.33	2.26	2.23	0.11
Monocyte (×10^3^ μl/L)	0.320	0.325	0.316	0.320	0.016
Neutrophils (×10^3^ μl/L)	22.9	23.0	22.9	22.9	1.1
Red blood cell (×10^12^/L)	3.89	3.85	3.86	3.87	0.19

Note

Means in a row with different superscript are significantly different (*p* < .05).

Abbreviation: *SEM*, standard error of the mean.

## 4 DISCUSSION

The substitution of noug cake and soybean meal by dried papaya pomace induced no negative effects on any of the tested parameters, and even outperformed the control diet in terms of laying performance, egg quality and hens metabolism. The blood metabolite profile can however not explain why performance with the highest inclusion level (7%) is falling back to the same level as the control group since none of the tested blood parameters become worse at the highest rate of inclusion.

Therefore, the inability to achieve an improved response of feed efficiency and laying rate as the level of inclusion advances may rather originate from slight imbalances in non‐formulated nutrients. Since no detailed information on papaya pomace was available for this study, such as digestible amino acid concentrations, future work should unravel the underlying mechanism. Papaya itself appears to be limiting in several amino acids (Marfo et al., 1986). This can explain that at a certain inclusion level, limiting amino acids start to reduce feed efficiency and laying performance (Khajali et al., 2008). However, a less efficient use of amino acids for protein synthesis would be reflected in increased serum uric acid concentrations, whereas the opposite was noted with increased papaya pomace inclusion. Therefore, the underlying reason for the failure of growing responses as a function of inclusion rate needs further investigation.

The stimulating effect on laying performance may come from the flavonoid compounds such as myricetin and kaempferol in papaya peels which have an estrogenic activity inducing productive performance of layers by regulating the hypothalamic gonadal axis (Novitasari et al., 2018). This has already been confirmed by the previous study using flaxseeds and/or fenugreek seeds for Bovans Brown layers (Saleh et al., 2019).

The unaltered eggshell quality indicates that papaya pomace use will not lead to higher egg breakage and had no notable effects on for instance calcium homeostasis. Literature data indeed confirm that papaya contains fair amounts of calcium (Emeka & Ojimelukwe, 2012).

The increased albumen height and Haugh score suggest a better storage potential for eggs from hens fed papaya pomace. A good level of antioxidant action is often responsible for good albumen quality, and here again, the natural presence of antioxidant flavonoids can support this hypothesis (Nugroho et al., 2017). The antioxidant action of papaya products has been widely documented in literature (Zhou et al., 2011), usually attributed to the high concentrations of carotenoids that cause the yellow colour of papaya (Chandrika et al., 2003). It is the logical reason why yolk colour was strongly affected by papaya pomace inclusion in the laying hens diet. Egg yolk colour is referred to be the major trait associated with consumer preference for table egg (Card et al., 1972). The improvement in yolk colour could be due to the presence of carotenoids such as β‐cryptoxanthin, lutein and β‐carotene in papaya pomace (Aroche et al., 2018); (AOAC, 2006). Similar to our findings, (Leke et al., 2018) reported that yolk colour intensity was significantly improved when papaya peel was fed to layers.

Serum biochemical parameters are the major indictors of metabolic alterations in tissue and organs reflecting diet effects on the nutrient metabolism and health of chickens (Bermudez and Stewart‐Brown, 2003). The reduction in serum total cholesterol and low density lipoprotein while high density lipoprotein was increased is in agreement with the results of (Leke et al., 2018) on papaya peels. The reduction of cholesterol in layers fed papaya pomace might be attributed to the presence of β‐sistosterol in papaya pomace which have hypoglycemic and hypolipidemic activity of impairing absorption of cholesterol in the small intestine (). The papaya pomace‐induced change in cholesterol fractions towards more HDL will likely have contributed to the increased egg laying rate, since HDL is needed to transport lipids for egg yolk synthesis to the ovaries (Alvarenga et al., 2011); (Andersen et al., 2013). The reduction of uric acid as the level of inclusion increases reflects a decreasing need to use protein as energy source. Although not significant, the numeric decreases in aspartate amino transferase and alanine amino transferase activities support this interpretation as their main function is to channel amino acids into the citric acid cycle (Huang et al., 2006). The above changes in metabolite profile are likely attributable to increased performance rather than altered health status, since none of the haematological parameters were affected in our study.

## 5 CONCLUSION

Substitution of noug meal and soybean meal by dried papaya pomace in laying hen diets showed a non‐linear improvement of feed efficiency and laying rate. This improvement in efficiency was further evidenced by the changes in lipid and cholesterol metabolism. The papaya pomace inclusion also induced an improved response in egg traits such as albumen height and yolk colour that are most likely due to the natural carotenoids. Dried papaya pomace is therefore a valuable by‐product to use in laying hen diets.

## CONFLICT OF INTEREST

The authors declare that they have no conflict of interest.

## AUTHOR CONTRIBUTION

**Bogale Tamiru:** Conceptualization; Investigation; Methodology. **Ashraf Alkhtib:** Conceptualization; Data curation; Methodology; Software; Writing‐original draft; Writing‐review & editing. **Metekia Tamiru:** Conceptualization; Data curation; Formal analysis; Investigation; Supervision; Writing‐original draft; Writing‐review & editing. **Solomon Demeke:** Conceptualization; Methodology; Supervision. **Emily Burton:** Writing‐original draft; Writing‐review & editing. **Taye Tolemariam:** Conceptualization; Methodology; Writing‐review & editing. **Lemessa Debela:** Methodology; Resources. **Geert Paul Jules Janssens:** Writing‐original draft; Writing‐review & editing.

## ETHICAL APPROVAL

The experiment received approval from the Ethical Committee of Jimma University, Ethiopia.

### PEER REVIEW

The peer review history for this article is available at https://publons.com/publon/10.1002/vms3.516.

## References

[vms3516-bib-0001] Al‐Sagan, A. A., AL‐Yemni, A. H., Al‐Abdullatif, A. A., Attia Y. A., & Hussein E. O. S. (2020). Effects of different dietary levels of blue lupine (*Lupinus angustifolius*) seed meal with or without probiotics on the performance, carcass criteria, immune organs, and gut morphology of broiler chickens. frontiers in veterinary. Science, 7, 1–14. 10.3389/fvets.2020.00124 PMC7082746 32232061

[vms3516-bib-0002] Alvarenga R. R., Zangeronimo M. G., Pereira L. J., Rodrigues P. B., & Gomide E. M. (2011). Lipoprotein metabolism in poultry. World’s Poultry Science Journal. 431–440.

[vms3516-bib-0003] Andersen, C. J., Blesso, C. N., Lee, J., Barona, J., Shah, D., Thomas, M. J., & Fernandez, M. L. (2013). Egg consumption modulates HDL lipid composition and increases the cholesterol‐accepting capacity of serum in metabolic syndrome. Lipids, 48(6), 557–567. 10.1007/s11745-013-3780-8 23494579 PMC3869568

[vms3516-bib-0004] AOAC . (2006). Official methods of analysis. AOAC: .

[vms3516-bib-0005] Aroche, R., Martínez, Y., Ruan, Z., Guan, G., Waititu, S., Nyachoti, C. M., Más, D., & Lan, S. (2018). Dietary inclusion of a mixed powder of medicinal plant leaves enhances the feed efficiency and immune function in broiler chickens. Journal of Chemistry, 2018, 1–6. 10.1155/2018/4073068

[vms3516-bib-0006] Ayhan, V., Arslan duru, A., & Özkaya, S. (2009). Kurutulmuş Elma Posasının Etlik Piliç Rasyonlarında Kullanım Olanakları. Kafkas Universitesi Veteriner Fakultesi Dergisi, 15(5), 669–672. 10.9775/kvfd.2009.077-A

[vms3516-bib-0009] Bermudez, & Stewart‐Brown. (2003) Principles of disease prevention diagnosis and control: Disease prevention and diagnosis. Diseases. In: Saif, Y. M., Barnes, H. J., Fadly, A., Glisson, J. R., Mcdougald, L. R., Swayne, D. E. (Eds). Iowa State University Press; 17–55.

[vms3516-bib-0010] Card, L. E., & M, C. N. (1972). Diseases and parasites. In: Lea, F. A. editors. Poultry Production. 11th ed. ; 1972. p. 244–273.

[vms3516-bib-0011] Chandrika, U. G., Jansz, E. R., Wickramasinghe, S. M. D. N., & Warnasuriya, N. D. (2003). Carotenoids in yellow‐ and red‐fleshed papaya (Carica papaya L). Journal of the Science of Food and Agriculture, 83(12), 1279–1282. 10.1002/jsfa.1533

[vms3516-bib-0012] Diarra, S. (2018). Chemical composition, dietary recommendations and prospects. Journal of Animal Physiology and Animal Nutrition, 102, 1284–1295. 30009539 10.1111/jpn.12954

[vms3516-bib-0014] Emeka N. G., Ojimelukwe P. (2012). Variability in proximate, mineral and vitamin contents of Carica papaya (L.) leaves, fruit pulp and seeds. International Journal of Medicinal and Aromatic Plants, 2, 90–96.

[vms3516-bib-0015] Emshaw, Y., Melesse, A., & Assefa, G. (2012). The effect of dietary inclusion of mango (Magnifera indica L.) fruit waste on feed intake, growth and feed efficiency of Cobb‐500 broiler. Chickens, 83, 73–83.

[vms3516-bib-0016] Gabrekiristos, E. (2020). A newly emerging disease of papaya in ethiopia: black spot (*Asperisporium caricae*) disease and management options. Journal of Plant Pathology & Microbiology, 1–5.

[vms3516-bib-0017] Gerzilov, V., Nikolov, A., Petrov, P., Bozakova, N., Penchev, G., & Bochukov, A. (2015). Effect of a dietary herbal mixture supplement on the growth performance, egg production and health status in chickens. Journal of Central European Agriculture, 16(2), 10–27. 10.5513/JCEA01/16.2.1580

[vms3516-bib-0018] Gha, V., Fisher, D., & Henkel, R. (2019). Carica papaya seed extract slows human sperm. Journal of Ethnopharmacology, 241, 111972. 10.1016/j.jep.2019.111972 31128152

[vms3516-bib-0019] Haselmeyer, A., Zentek, J., & Chizzola, R. (2014). Effects of thyme as a feed additive in broiler chickens on thymol in gut contents, blood plasma, liver and muscle. Journal of the Science of Food and Agriculture, 95(3), 504–508. 10.1002/jsfa.6758 24862829

[vms3516-bib-0020] Huang, X.‐J., Choi, Y.‐K., Im, H.‐S., Yarimaga, O., Yoon, E., & Kim, H.‐S. (2006). Aspartate Aminotransferase (AST/GOT) and Alanine Aminotransferase (ALT/GPT) detection techniques. Sensors, 6(7), 756–782 10.3390/s6070756

[vms3516-bib-0022] Jain, N. S. (1986). Veterinary haematology.

[vms3516-bib-0023] Kamaruzzaman, M., Chowdhury, S. D., Podder, C. K., & Pramanik, M. A. H. (2005). Dried papaya skin as a dietary ingredient for broiler chickens. British Poultry Science, 46, 390–393. 10.1080/00071660500126631 16050195

[vms3516-bib-0024] Kasapidou, E., Sossidou, E., & Mitlianga, P. (2015). Fruit and vegetable co‐products as functional feed ingredients in farm animal nutrition for improved product quality. Agriculture, 5(4), 1020–1034. 10.3390/agriculture5041020

[vms3516-bib-0025] Khajali, F., Khoshouie, E. A., Dehkordi, S. K., & Hematian, M. (2008). Production performance and egg quality of Hy‐Line W36 laying hens fed reduced‐protein diets at a constant total sulfur amino acid: Lysine ratio. Journal of Applied Poultry Research, 17(3), 390–397. 10.3382/japr.2008-00002

[vms3516-bib-0026] Leke, J. R., Sompie, F. N., Wantasen, E., & Tallei, T. E. (2018). Nutritional characteristics and quality of eggs from laying hens fed on papaya peel meal diet. Animal Production, 20(32), 147–154.

[vms3516-bib-0027] Lira, R. C., Rabello, C. B., Ferreira, P. V., Lana, R. Q., Lüdke, J. V., & Dutra Junior, W. M. (2009). Inclusion of guava wastes in feed for broiler chickens. Revista Brasileira De Zootecnia, 38(12), 2401–2407. 10.1590/S1516-35982009001200016

[vms3516-bib-0028] Marfo, E., Oke, O., & Afolabi, O. (1986). Some studies on the proteins of Carica papaya seeds. Food Chemistry, 22(4), 267–277 10.1016/0308-8146(86)90085-3

[vms3516-bib-0029] Martínez, Y., Valdivie, M., Martínez, O., Estarrón, M., & Córdova, J. (2010). Utilization of pumpkin (Cucurbita moschata) seed in broiler chicken diets. Cuban Journal of Agricultural Science, 44(4), 387–392.

[vms3516-bib-0031] Navid, J., Mozaffar, M., & Kazem, K. (2014). Effect of dietary medicinal herbs on performance, egg quality and immunity response of laying hens. Advances in Environmental Biology, 7(13), 4382–4389.

[vms3516-bib-0032] Novitasari, D., Mada, U. G., Arifah, F. H., Mada, U. G., Murwanti, R., & Mada, U. G. (2018). Estrogenic activity of ethanolic extract of papaya peels (Carica Papaya L.) on uterine weight and mammae gland proliferation on ovariectomy rats. Indonesian Journal of Cancer Chemoprevention, 9(2), 86–91. 10.14499/indonesianjcanchemoprev9iss2pp86-91

[vms3516-bib-0033] Nugroho, A., Heryani, H., Choi, J. S., & Park, H. J. (2017). Identification and quantification of flavonoids in Carica papaya leaf and peroxynitrite‐scavenging activity. Asian Pacific Journal of Tropical Biomedicine, 7(3), 208–213.

[vms3516-bib-0034] Pavithra, C. S., Devi, S. S., Suneetha, W. J., & Rani, C. V. D. (2018). Development and evaluation of papaya peel powder and paste incorporated chapathis. Journal of Pharmaceutical Research International, 24(1), 1–5. 10.9734/JPRI/2018/41384

[vms3516-bib-0035] Rama Rao, S. V., Raju, M. V. L. N., Panda, A. K., & Reddy, M. R. (2006). Sunflower seed meal as a substitute for soybean meal in commercial broiler chicken diets. British Poultry Science, 47(5), 592–598. 10.1080/00071660600963511 17050104

[vms3516-bib-0036] Saki, A. A., Aliarabi, H., Hosseini Siyar, S. A., Salari, J., & Hashemi, M. (2014). Effect of a phytogenic feed additive on performance, ovarian morphology, serum lipid parameters and egg sensory quality in laying hen. Veterinary Research Forum: An International Quarterly Journal, 5(4), 287–293. 25610580 PMC4299994

[vms3516-bib-0037] Saleh, A. A., Ahmed, E. A. M., & Ebeid, T. A. (2019). The impact of phytoestrogen source supplementation on reproductive performance, plasma profile, yolk fatty acids and antioxidative status in aged laying hens. Reproduction in Domestic Animals, 54(6), 846–854. 10.1111/rda.13432 30916364

[vms3516-bib-0038] Saleh, A. A., Ragab, M. M., Ahmed, E. A. M., Abudabos, A. M., & Ebeid, T. A. (2018). Effect of dietary zinc‐methionine supplementation on growth performance, nutrient utilization, antioxidative properties and immune response in broiler chickens under high ambient temperature. Journal of Applied Animal Research, 46(1), 820–827. 10.1080/09712119.2017.1407768

[vms3516-bib-0039] Saleh, A. A., Zaki, A., El‐Awady, A., Amber, K., Badwi, N., Eid, Y., & Ebeid, T. A. (2020). The effect of substituting wheat bran with cumin seed meal on laying performance, egg quality characteristics and fatty acid profile in laying hens. Veterinarski Arhiv, 90(1), 47–56. 10.24099/vet.arhiv.0500

[vms3516-bib-0041] SAS. SAS/STAT 12.1 user’s guide. SAS Inc. 2012.

[vms3516-bib-0042] Setargie, A., Mekbib, F., & Abraha, E. (2015). In vitro propagation of papaya (Carica papaya L.), World Journal of Agricultural Sciences, 11(2), 84–88.

[vms3516-bib-0043] Singh, O., & Ali, M. (2011). Phytochemical and antifungal profiles of the seeds of Carica papaya L. Indian Journal of Pharmaceutical Sciences, 73(4), 447–909. 22707832 10.4103/0250-474X.95648 PMC3374564

[vms3516-bib-0044] Sola‐ojo, F. E., & Bolu, S. (2009). Effect of graded levels of dried pawpaw (Carica papaya) seed on the performance, haematology. Serum Biochemistry and Carcass Evaluation of Chicken Broilers, 8(9), 905–909.

[vms3516-bib-0045] Swiatkiewicz, S., Arczewska‐Wlosek, A., & Józefiak, D. (2016). The use of cottonseed meal as a protein source for poultry: An updated review. World’s Poultry Science Journal, 72(3), 473–483. 10.1017/S0043933916000258

[vms3516-bib-0046] Wiseman, J. (1987). Feeding of non‐ruminant livestock. Butterworth and Co.

[vms3516-bib-0047] Zhou, K., Wang, H., Mei, W., Li, X., Luo, Y., & Dai, H. (2011). Antioxidant activity of papaya seed extracts. Molecules, 16(8), 6179–6192. 10.3390/molecules16086179 21788927 PMC6264420

